# Antisense *yycG* modulates the susceptibility of *Staphylococcus aureus* to hydrogen peroxide via the *sarA*

**DOI:** 10.1186/s12866-021-02218-x

**Published:** 2021-05-30

**Authors:** Shizhou Wu, Yunjie Liu, Lei Lei, Hui Zhang

**Affiliations:** 1 Department of Orthopedics, West China Hospital, Sichuan University, No.37 Guoxue Alley, Sichuan 610041 Chengdu, P.R. China; 2 West China School of Public Health, Sichuan University, Chengdu, China; 3 Department of Preventive Dentistry, Hospital of Stomatology, State Key Laboratory of Oral Diseases,, Sichuan University, NO.14 Third Section, Renmin South Road, Sichuan 610041 Chengdu, P.R. China

**Keywords:** Antisense, YycFG, Hydrogen peroxide, *Staphylococcus aureus*, *sarA*

## Abstract

**Background:**

The infectious pathogen *Staphylococcus aureus* (*S. aureus*) is primarily associated with osteomyelitis. Hydrogen peroxide drainage is an effective antimicrobial treatment that has been adopted to combat *S. aureus* infections. Previous investigations have indicated that the antisense RNA (asRNA) strategy negatively modulates *S. aureus* YycFG TCS, and it significantly disrupts biofilm formation. However, the effects of the antisense *yycG* RNA (AS*yycG*) strategy on the susceptibility of biofilm-producing *S. aureus* to hydrogen peroxide and the mechanisms underlying this effect have not been elucidated to date.

**Results:**

Overexpression of AS*yycG* inhibited the transcription of biofilm formation-related genes, including *sarA* and *icaA*. Additionally, the CFU counts and the live bacterial ratios of AS*yycG* biofilm-producing *S. aureus* treated with H_2_O_2_ were notably reduced across the groups. Notably, the predicted promoter regions of the *sarA* and *icaA* genes were directly regulated by YycF.

**Conclusions:**

AS*yycG* was observed to sensitize biofilm-producing *S. aureus* to H_2_O_2_ intervention synergistically via the *sarA* and thus may represent a supplementary strategy for managing osteomyelitis. However, future in-depth studies should attempt to replicate our findings in animal models, such as the rat osteomyelitis model.

**Supplementary Information:**

The online version contains supplementary material available at 10.1186/s12866-021-02218-x.

## Background

In humans, *Staphylococcus aureus* (*S. aureus*) is associated with a variety of diseases, ranging from relatively mild skin and soft tissue infections to life-threatening bacteremia and endocarditis. Hydrogen peroxide drainage is an effective antimicrobial treatment to combat *S. aureus* colonization and invasion [1]. Notably, it is well-known that 1000-fold greater resistance to antimicrobial agents was observed in the biofilm state than in the planktonic state [2–5]. During the establishment of biofilms, polysaccharide intercellular adhesion (PIA) is considered to be the key mechanism that provides an essential scaffold for biofilms and a special kind of extracellular matrix that is encoded by the *ica* operon and *sarA* [6, 7]. The *sarA* gene has been reported to drive biofilm formation by altering *ica* transcription and producing PIA [2, 4]. In *Staphylococcus epidermidis* (*S. epidermidis*), SarA was reported to be an essential positive regulator of the *ica* operon in biofilm development [8]. Additionally, numerous studies have evaluated the regulatory pathway of the *sarA* gene and demonstrated that *sarA* was associated with bacterial oxidation sensing, virulence factors, and biofilm formation in *S. aureus* [9–11].

Two-component signal transduction systems (TCSs) are essential regulators of staphylococcal metabolism and adaptation to environmental changes [12, 13]. Among the 16 known TCSs in *S. aureus*, YycFG is essential for bacterial viability [14]. The YycG histidine kinase is generally anchored by a cytoplasmic membrane and is involved in monitoring environmental changes. Notably, when the responses of this kinase to extracellular stimuli transfer the phosphoryl group to activate the response regulator (RR) YycF, the cellular physiology status, including biofilm organization, adapts to changes [7, 14, 15]. In a previous study, we demonstrated that the essential YycFG TCS was closely related to biofilm formation and extracellular matrix organization [16]. However, since the YycFG TCS is essential to *S. aureus* viability, the construction of deletion mutants of YycFG was unsuccessful [17].

Antisense RNA (asRNA) is a single-stranded RNA that is complementary to the target messenger RNA (mRNA). The interaction between the RNA molecules inhibits downstream signal transduction activation. Using this asRNA strategy, we constructed mutant antisense *S. aureus* strains that express YycFG TCS at low levels. The number of *icaA* gene transcripts and the level of biofilm formation decreased in the AS*yycG*-mutant *S. aureus*, and YycFG was expressed at low levels [18]. However, the potential mechanisms by which the transcriptional regulator YycF modulates *sarA* and/or *icaA* expression and the biological effects of this modulation on the susceptibility of biofilm-producing *S. aureus* to hydrogen peroxide warrant further research.

In the present study, we investigated the role played by antisense *yycG* in regulating the susceptibility of biofilm-producing *S. aureus* to H_2_O_2_ treatment. In addition, the potential association of YycF with *sarA* and *icaA* was evaluated using an electrophoretic mobility shift assay (EMSA). This work primarily investigated whether an antisense *yycG* interference strategy could sensitize biofilm-producing *S. aureus* to H_2_O_2_ intervention, which may be associated with the direct regulation of YycF to the adjacent genes *sarA* and *icaA*.

## Results

### Antisense *yycG* modulated oxidative regulation

**Fig. 1:**
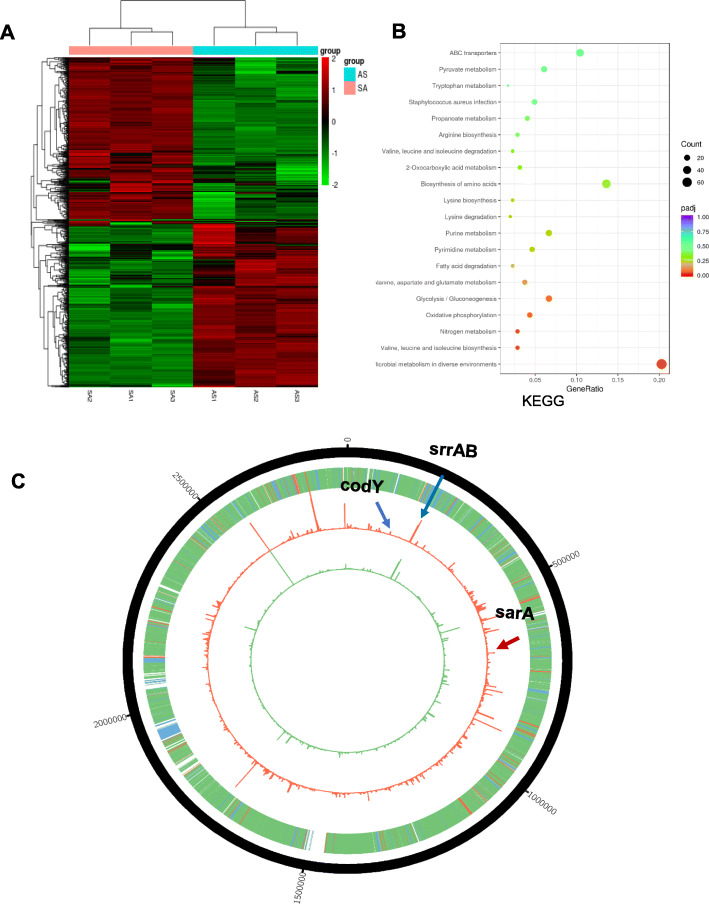
Transcriptome analysis of the antisense *yycG *modulation. **a** Heatmap for the Transcriptome analysis;** b **KEGG annotation statistics; **c** Genomic map showing transcription profiles of ATCC29213 and antisense *yycG *mutant strains. The middle (red) circle depicts FPKM values for antisense *yycG* mutant cells, and the inner (green) circle depicts FPKM values for the ATCC29213 strains. The outer (yellow) circle is a heat map showing fold changes in expression between the ATCC29213 and the antisense *yycG* mutant

Transcriptome and enrichment analyses showed that overexpression of antisense *yycG* influenced the pathways associated with biofilm metabolism, virulence, oxidative regulation, and glycolysis/gluconeogenesis utilization by *S. aureus* (Fig. 1a and b). Notably by padj values ranking, the 20 KEGG pathways with the greatest differential expression that were involved in *S. aureus* infection and metabolism were determined to have enrichment score mostly at around 0.5 differences between the antisense *yycG* overexpression group and ATCC29213 group. The less padj value was, the greater expression diversity in pathway between the antisense *yycG* overexpression group and ATCC29213 group. Each pathway involved different genes count, such as microbial metabolism in diverse environments involving about 60 genes with a padj value at about 0.05 (Fig. 1b). Moreover, three genes that regulate biofilm formation and virulence were modulated; specifically, *sarA* was downregulated, and *codY* and *srrA* were upregulated (see Fig. 1c). These results indicated that antisense *yycG* negatively regulated biofilm formation and the expression of the virulence-associated gene *sarA.*

### Antisense *yycG* sensitized biofilm-producing *S. aureus* to H_2_O_2_ intervention synergistically

**Fig. 2:**
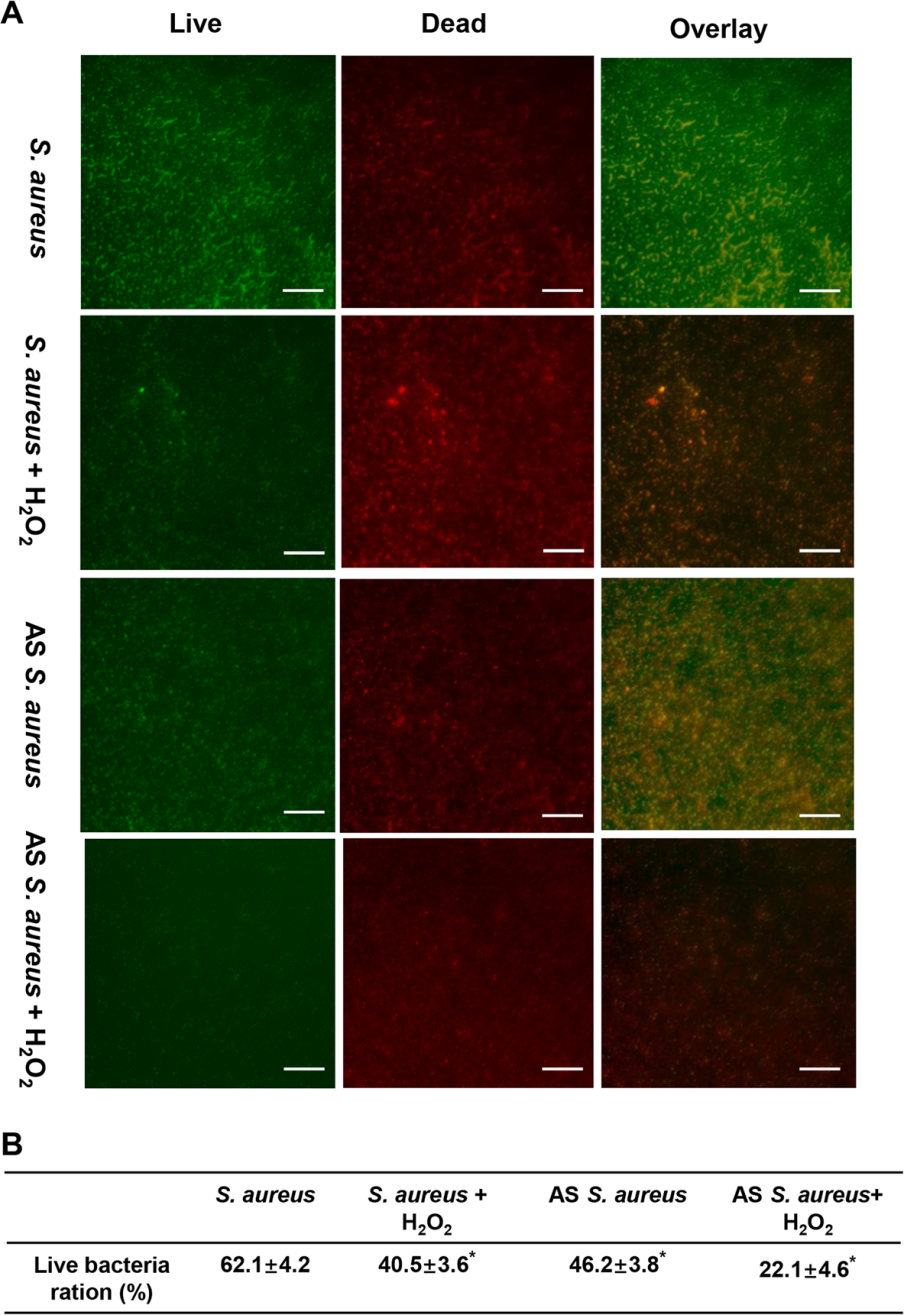
The AS *yycG* increased susceptibility of the *S. aureus* to H_2_O_2_. **a** *S. aureus*, and AS*yycG* strains after H_2_O_2_ treatment. Green, viable bacteria (SYTO 9); red, dead bacteria (PI); scale bars, 100 μm; **b** Percentage (%) of viable *S. aureus* cells after H_2_O_2_ treatment (n = 10, *P < 0.05)

**Fig. 3:**
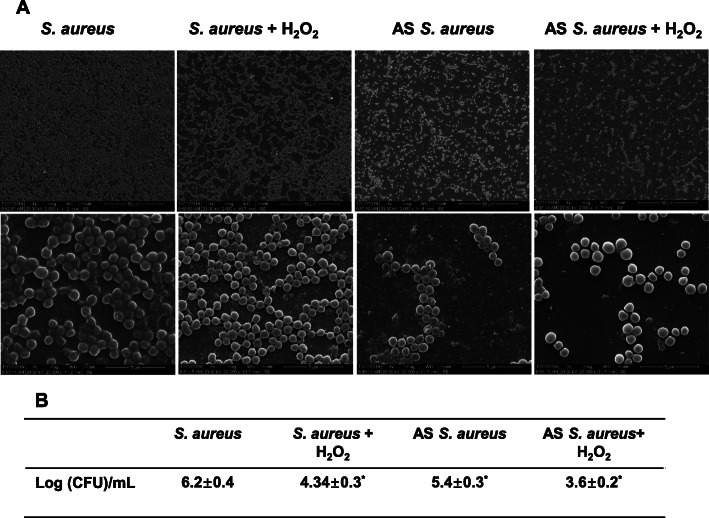
The AS *yycG* sensitized biofilm producing *S. aureus* to H_2_O_2_ intervention synergistically. **a** SEM images of *S. aureus*, and AS*yycG* strains after H_2_O_2_ treatment; **b** Number of CFUs before and after the H_2_O_2_ treatment [*n* = 10, *P < 0.05, lg (CFU/mL)]

**Fig. 4:**
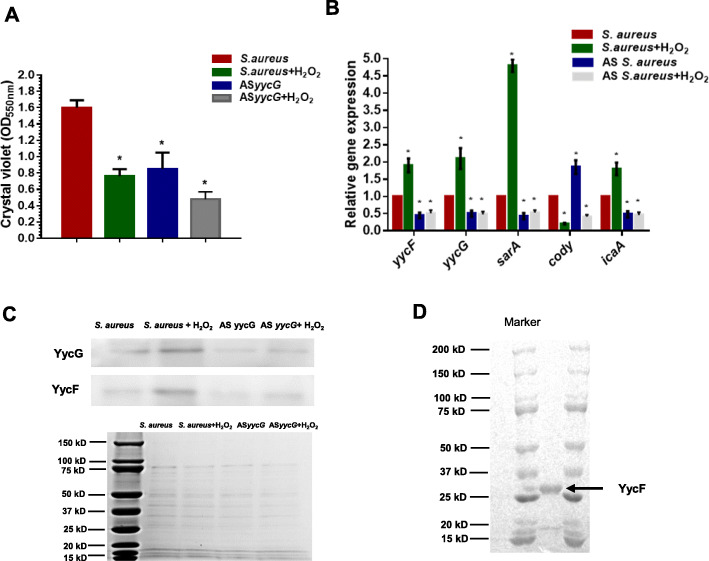
The AS *yycG* overexpression inhibited the transcripts of biofilm-related genes. **a** Biomass was quantified by crystal violet staining. Optical densities at 600 nm were measured (*n* = 10, ^*^P < 0.05); **b** Quantitative RT-PCR analysis showed the gene transcripts in *S. aureus*, and AS*yycG* strains treated with H_2_O_2_. *S. aureus* gene expression was relatively quantified by RT-PCR using 16 S as an internal control (*n* = 10, ^*^P < 0.05); **c** The productions of YycF and YycG were quantified in the cells of *S. aureus*, and AS*yycG* strains treated with H_2_O_2_ for Western blotting (upper lane). The lower panel shows a Coomassie-stained gel supporting equal loading of the samples for total bacterial lysis; **d** The purified recombinant YycF protein was visualized by Coomassie staining after SDS-PAGE

Through fluorescence microscopy, the live ratios of bacteria were compared. In the AS*yycG +* H_2_O_2_ group (Fig. 2a), the antibacterial effect of H_2_O_2_ was significantly enhanced by AS*yycG* synergistically and exhibited the lowest live bacteria ratio at 22.1 ± 4.6 compared with each group (Fig. 2b). The SEM and immunofluorescence results were similar and exhibited the lowest EPS levels in the AS*yycG +* H_2_O_2_ group. In addition, we could only detect rare cell clusters in AS*yycG* + H_2_O_2_, even after 24 h of culture (Fig. 3a). Quantitively, we performed a CFU test on each biofilm. Correspondingly, the CFU count of AS *S. aureus* + H_2_O_2_ biofilm was mostly lower in all groups than in the *S. aureus* ATCC29213 parent group (Fig. 3b). Accordingly, we evaluated the ability of the *S. aureus* strains to form biofilms. The biomass was quantified via a microtiter dish assay, and AS*yycG* strains exhibited reduced biofilm formation compared with the *S. aureus* ATCC29213 group. In particular, the AS*yycG* strains treated with H_2_O_2_ exhibited the strongest decrease in biofilm formation among all groups, which indicated that AS *S. aureus* + H_2_O_2_ biofilms had significantly decreased biofilm growth compared to the other groups (Fig. 4a). These results showed that antisense *yycG* could significantly enhance the susceptibility of *S. aureus* to H_2_O_2_ and exert negative effects on biofilm-producing *S. aureus* after H_2_O_2_ treatment.

### Antisense *yycG* overexpression inhibited the transcription of the *sarA* and *ica *genes

Quantitative RT-PCR analyses demonstrated that the expression levels of the genes in the YycFG pathway, including *yycG* and *yycF*, were significantly reduced, which may be attributable to the overexpression of AS*yycG* in the AS*yycG* group treated with H_2_O_2_. Furthermore, the expression levels of *sarA* related to the oxide stress reaction and biofilm-associated *ica* genes were more significantly reduced by AS*yycG* overexpression in the AS*yycG* strains treated with H_2_O_2_ than in the *S. aureus* parent ATCC29213 strains (n = 10, *P* < 0.05; Fig. 4b). In keeping with this result, YycFG pathway protein expression was significantly downregulated in the AS*yycG* group treated or not treated with H_2_O_2_ compared with the ATCC29213 strain. Notably, the protein expression levels of the cognate sensor kinase YycG and the response regulator YycF were elevated in response to H_2_O_2_ intervention (Fig. 4c). These results indicated that antisense *yycG* inhibited the expression of the YycFG pathway, which may have contributed to reductions in downstream *icaA* and *sarA* gene expression.

### YycF bound to the predicted promoter regions of *sarA* and *icaA *genes

**Fig. 5:**
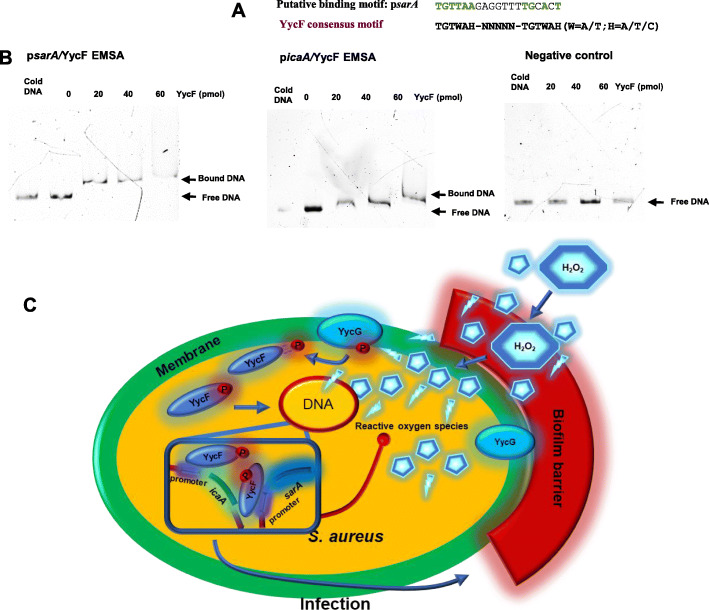
EMSA for *sarA* and *icaA* promoter regions. **a** Consensus YycF binding motif and candidate sequences in promoters of *sarA*. TGTWAH-NNNNN-TGTWAH, where W is A/T and H is A/T/C; **b** EMSA in which promoter regions were obtained by PCR and FAM-labelled. Excess cold (unlabeled) promoter DNAs were used to out-compete YycF- specific binding. As the negative control, a DNA fragment the same size as the promoter region and similar AT: GC mole ratio, but missing the YycF consensus binding sequence, was used to rule out non-specific binding; **c** The working model of oxidation regulation by AS*yycG* via *sarA* pathway

The purified recombinant YycF protein was visualized via Coomassie staining after SDS-PAGE (Fig. 4d). The promoter region of *sarA* contained a putative YycF binding consensus motif (Fig. 5a). We employed the bacterial promoter database (http://www.softberry.com/berry.phtml?topic=index&group=programs&subgroup=gfindb), and a putative promoter of the *sarA* gene was predicted. EMSA demonstrated that the YycF protein was bound to DNA fragments from *sarA* and *icaA* promoter regions. As a negative control, to rule out nonspecific binding, we utilized a DNA fragment of the same size as the predicted promoter and with a similar AT:GC mole ratio but missing the YycF consensus binding sequence (Fig. 5b). By EMSAs, we determined that YycF could directly bind to the promoter regions of the *sarA* and *icaA* genes and regulate their expression, which contributed to biofilm metabolism and resistance to antibacterial agents, such as H_2_O_2_ (Fig. 5c).

## Discussion

Microbial biofilm formation is a principal factor for chronic infectious diseases, such as osteomyelitis; therefore, numerous antibiofilm strategies have been devised for clinical use by surgeons, such as aggressive irrigation and physical removal of infective debris [19, 20]. In addition, numerous other promising antibiofilm agents, such as zinc oxide nanoparticles (ZnO NPs), proteinase K, hamamelitannin (HAM) and antimicrobial peptides (AMPs), have also been developed [21, 22].

Although 3 % H_2_O_2_ is conventionally applied in most bone infection cases to disinfect microorganisms from wounds in clinical practice, the killing ability of H_2_O_2_ on pathogenic bacteria is doubtful [23–25], especially under biofilm conditions in which the resistance increases 1000-fold compared to planktonic bacterial conditions [26–29]. Biofilms, as a physical barrier, could protect microorganisms from the diffusion of environmental stimuli and confer antibiotic resistance to the microorganisms embedded within the biofilm. Multiple factors, including the existence of metabolically inactive bacterial and immune system evasion, contribute to the resistance of biofilms to disinfectant agents [30]. In this study, transcriptome analysis demonstrated that antisense *yycG* overexpression downregulated the expression of the oxidative reaction-, virulence- and biofilm-associated genes *ica* and *sar* in *S. aureus*, which may improve the susceptibility of *S. aureus* to H_2_O_2_.

Extracellular polysaccharide substance (EPS), an essential component of biofilms, plays an important role in preventing bacterial colonies from antimicrobial effects. In a previous study, improved killing efficiency of antimicrobial agents for bacteria embedded in EPS could be obtained by combined application with biofilm-dispersing enzymes [28, 31]. Our study obtained similar results, which showed that the lowest EPS levels and the fewest cell colony clusters were observed by SEM after 24 h of culture in the AS*yycG* + H_2_O_2_ group. The *icaADBC* operon synthesized polysaccharide intercellular adhesion (PIA) as a significant component of EPS in biofilm production, which is modulated by the only essential TCS, YycFG, to promote cell wall and biofilm metabolism [32]. A plasmid overexpressing a complementary base-pairing antisense YycG (AS*yycG*) can significantly reduce biofilm formation by inhibiting the YycFG pathway [33]. Notably, when EPSs are disorganized, the resultant exposure and release of residual biofilm cells to these agents enhance their sensitivity.

Furthermore, the CFU counting assays and live bacterial ratios indicated that *yycG* sensitized biofilm-producing *S. aureus* to H_2_O_2_ treatment synergistically. When we combined AS*yycG* with H_2_O_2_ antibacterial agents, not only was the antibacterial effect of H_2_O_2_ improved, but a prolonged inhibited biofilm formation effect was also acquired 24 h after treatment with low potential to cause a chronic infection. *In vivo* research would further be considered to evaluate the synergistic antibacterial effects of AS*yycG* combined with H_2_O_2_. Although 3 % H_2_O_2_ is continually applied to disinfect wounds microorganisms in clinical irrigation, it oxidizes protein, nucleic acid, and lipids of normal healthy cells, which limited the beneficial effect of promoting wound healing becomes [23, 24]. However, at comparatively low concentrations, the killing ability of H_2_O_2_ on pathogenic bacteria is doubtful [24, 25]. In the present study, the application of antisense *yycG* downregulated the expression of the EPS synthesis-associated gene *ica*, inhibited biofilm formation and enhanced susceptibility to H_2_O_2_ in *S. aureus*. From this perspective, with the supplementary antisense *yycG*, an appropriate low therapeutically concentration of H_2_O_2_ could be apply to maintain this balance by enhancing antibacterial effect and keeping wound healing with ameliorating wound redox extent.

On the other hand, the *sarA* gene was reported to enhance biofilm formation by altering *ica* transcription and the production of PIA in *S. aureus* [2, 4]. In *S. epidermidis*, SarA was demonstrated to be an essential positive regulator of the *ica* operon in biofilm development [8]. In addition, homologous YycF could directly regulate *icaA* and *sarA* gene expression in *S. epidermidis* [7]. In the present study, the promoter regions of *sarA* were directly bound by YycF, as demonstrated via EMSA, which showed that YycF could indirectly regulate *ica* through the *sarA* pathway.

Regarding environmental stimuli, the essential TCS YycFG (also known as WalRK) was reported to be associated with bacterial oxidation sensing [34, 35]. Notably, in the present study, the protein production of the cognate sensor kinase YycG, as well as the response regulator YycF, was enhanced in response to H_2_O_2_ intervention, which again confirms that TCS YycFG can be applied to monitor environmental changes. By oxidative stress, biofilm-producing *S. aureus* and the expression of biofilm-inducing genes, such as *sarA*, were supposed be elevated [35]. However, the expression levels of the antioxidant stress reaction-related and biofilm-associated *sarA* and *ica* genes were significantly reduced by AS*yycG* RNA in the AS*yycG* strains treated with H_2_O_2_ compared with the parental *S. aureus* ATCC29213 strains in our study. In addition, the global regulator CodY controls the expression of dozens of metabolism and virulence genes in *S. aureus*, including genes associated with biofilm formation. The activity of CodY in regulating biofilm formation and cell aggregation was associated with *ica* expression and PIA production [36]. CodY, a regulatory protein, has been reported to repress virulence gene expression in *S. aureus* [37]. In present study the expression of CodY was relatively high in AS*yycG* group, which may contribute to repress the virulence gene expressions in *S. aureus*. However, whether CodY is involved in sensing bacterial oxidation warrants further investigation, particularly concerning the mechanisms by which it regulates virulence genes. Additionally, the regulatory effect of the *srrA* gene associated with the YycFG TCS merits further investigation.

## Conclusions

The effects of the antisense *yycG* RNA strategy on the susceptibility of biofilm-producing *S. aureus* to H_2_O_2_ treatment were investigated. Recombinant shuttle plasmids were used to overexpress an antisense *yycG* RNA (AS*yycG*) and were transformed into the *S. aureus* strain to construct AS*yycG S. aureus*. The results of this study indicated that *yycG* overexpression inhibited the transcription of biofilm formation-related genes, including *sarA* and *icaA*. Additionally, the CFU counts of AS*yycG* biofilms treated with H_2_O_2_ were mostly decreased. Notably, YycF could bind to the predicted promoter regions of *sarA* and *icaA* genes, demonstrating that susceptibility of biofilm-producing *S. aureus* to H_2_O_2_ intervention may be regulated by AS*yycG* via the *sarA* and *icaA* pathways. In summary, these results indicate that AS*yycG* synergistically sensitizes biofilm-producing *S. aureus* to H_2_O_2_ treatment and thus may be considered a supplementary strategy for managing osteomyelitis. Considering the possibility of antisense *yycG* breakdown by H_2_O_2_, a stabilizing vector, such as nanographene oxide, may be utilized in future research to support the clinical strategy of combining H_2_O_2_ with antisense *yycG*.

## Methods

### Bacterial strains and biofilms growth conditions

*S. aureus* strain ATCC29213, provided by the Department of Laboratory Medicine (West China Hospital, Sichuan University, Chengdu, China), was cultured in tryptic soy broth (TSB) [16]. In a previous study, we performed phenotypic experiments to ensure that *S. aureus* strain ATCC29213 was a biofilm-producing pathogen [33]. After overnight incubation at 37 °C and 5 % CO_2_, 500 µL of *S. aureus* suspension was inoculated into 10 mL fresh TSB medium to mid-logarithmic phase (optical density at 600 nm [OD_600_] = 0.5). For the formation of biofilms, sterilized glass disks (diameter of 10 mm) were dropped into log-phased *S. aureus* sterile 24-well microtiter plates for 24-h coculture biofilms. This basic culture section was prepared for the subsequent tests.

### Construction of *S. aureus* antisense *yycG* overexpression strains

To observe the effect of antisense *yycG* in *S. aureus*, we first constructed antisense *yycG*-overexpressing *S. aureus* strains according to the following protocols. Recombination of the plasmid pDL278 containing the antisense *yycG* fragment was conducted by inserting the antisense *yycG* (AS*yycG*) sequence into restriction sites between BamHI and EcoRI, which was subsequently synthesized by Sangon Biotech (Shanghai, China). According to our previous protocol [38], an AS*yycG*-overexpressing *S. aureus* (AS*yycG* mutants) was constructed by adding 200 ng recombinant pDL278 AS*yycG* plasmid with 1 µg/mL competence-stimulating peptide (CSP) into a 250-µL mid-exponential-phase *S. aureus* suspension for 60 min [33]. The empty pDL278 plasmid did not exert any effects on the viability of *S. aureus* [39].

### RNA extraction analyses

To investigate the alterations in the whole transcriptome between *S. aureus* ATCC29213 and AS*yycG* mutants, whole transcriptome analysis was applied. First, samples of *S. aureus* strain ATCC29213 and AS*yycG* mutants were cultured in 10 mL of fresh TSB media into the mid-logarithmic phase. Total RNA was isolated and quantified as previously described [33]. Agarose gel electrophoresis (1 %) was run to check for degradation or contamination of the RNA. We used a NanoPhotometer® spectrophotometer (IMPLEN, CA, USA) for RNA quality and quantity checks. The integrity of the extracted RNA sample was assessed using the RNA Nano 6000 Assay Kit of the Bioanalyzer 2100 system (Agilent Technologies, CA, USA).

### Library preparation for whole transcriptome sequencing

After the abovementioned RNA extraction, exactly 5 µg RNA per sample for the *S. aureus* ATCC29213 and AS*yycG* mutant groups was used as input for library preparation. Sequencing libraries were generated using the NEBNext® Ultra™ RNA Library Prep Kit for Illumina® (NEB, USA) following the manufacturer’s recommendations. Next, index codes were added to attribute sequences to each sample. The rRNA was removed using a specialized kit. Fragmentation was performed using divalent cations under elevated temperature in NEBNext First Strand Synthesis Reaction Buffer (5X). Using a fragmented mRNA template, first-strand cDNA was synthesized using random hexamer primers in the M-MuLV Reverse transcription system. The RNA was digested by RNase H, and then second strand cDNA synthesis was subsequently performed using DNA Polymerase I and RNase H. The remaining overhangs were converted into blunt ends via exonuclease/polymerase activities. After adenylation of the 3’ ends of DNA fragments, NEBNext adapter with a hairpin loop structure was ligated. Next, USER Enzyme (NEB, USA) was employed to degrade the second strand of cDNA. The library fragments were purified using the AMPure XP system (Beckman Coulter, Beverly, USA) to select cDNA fragments with a length of 250 ~ 300 bp. Then, PCR was performed using Phusion High-Fidelity DNA polymerase, universal PCR primers, and Index (X) Primer. Similarly, the above PCR products were purified (AMPure XP system), and library quality was assessed on the Agilent Bioanalyzer 2100 system. The cluster generation of the index-coded samples was performed on a cBot Cluster Generation System using TruSeq PE Cluster Kit v3-cBot-HS (Illumina) according to the manufacturer’s instructions. After clustering, sequencing was performed on an Illumina NovaSeq platform, whereby 150-bp paired-end reads were generated.

### Differential expression analysis

Following the above library preparation, differential expression between the two groups was analyzed. In this analysis, high-quality clean data were obtained by eliminating reads with adapters and low-quality reads from raw data. Bowtie2 was applied to map the above-filtered reads to the reference genome. To quantify the gene expression level, HTSeq v0.6.1 was used to count the read numbers mapped to each gene. Next, the number of fragments per kilobase of transcript sequence per million base pairs sequenced (FPKM) of each gene was calculated. Differential expression analysis of two groups was performed using the DESeq R package (1.18.0) [40]. DESeq provided statistical routines for determining differential expression in digital gene expression data using the negative binomial distribution-based model. The resulting *P*-values were adjusted using Benjamini and Hochberg’s approach to control the false discovery rate. Genes with adjusted *P*-values < 0.05 found via DESeq were classified as differentially expressed.

### KEGG enrichment analysis of differentially expressed genes

After the procedure described above was performed, raw data regarding differentially expressed genes were acquired. To identify the functions of differentially expressed genes between the *S. aureus* and AS*yycG* groups, Kyoto Encyclopedia of Genes and Genomes (KEGG) enrichment analysis was applied. KEGG is a database resource for elucidating high-level functions and utilities of biological systems, such as cells, organisms, and ecosystems, from molecular-level information, especially large-scale molecular datasets generated by genome sequencing and other high-throughput experimental technologies (http://www.genome.jp/kegg/) [41]. In this study, we used KOBAS software to test the statistical enrichment of differentially expressed genes in KEGG pathways. The results showed 20 KEGG pathways with adjusted P-values of approximately 0.5, involving the *S. aureus* infection pathway, and enrichment score differences were detected between the antisense *yycG* overexpression group and ATCC29213 group.

### Bacterial exposure to oxidants

After genetic alternation analysis and associated functional enrichment screening, we examined biofilm characteristics associated with resistance strength to chemical situlas, particularly H_2_O_2_, by overexpressing AS*yycG.* To determine the susceptibility of *S. aureus* ATCC29213 and AS*yycG* mutants to oxidants, 24-h-old biofilms derived from these groups were treated with 3 % H_2_O_2_ (for clinical irrigation, H_2_O_2_ is usually 3 %) for 30 min at 37 °C. Residual H_2_O_2_ was removed by diluting biofilm samples in PBS three times [23, 42]. The biofilm sample disks were cultured in TSB medium for 24 h after treatment.

### Microtiter dish assay and colony-forming unit (CFU) counting

The microtiter dish assay was applied to examine the biomass of 24-h-old biofilms after interventions with crystal violet (CV). For the formation of biofilms, samples of *S. aureus* strain ATCC29213 and AS*yycG* mutants were cultured into the midlogarithmic phase in 1 mL TSB media, which were dropped into sterile 24-well microtiter plates for 24 h. After 24 h of culture, the culture medium was removed. We washed the established biofilms three times with PBS solution. The dye bound to the biofilms was assayed after 0.1 % (w/v) crystal violet staining for 15 min followed by 1 mL destaining solution (ethanol/acetone = 8:2). Subsequently, 100 µL of the solution was transferred into a new 96-well plate, and the absorbance was measured with a microplate reader (ELX800, Gene) at OD_600_ nm [33].

For the CFU counting test, the treated biofilms were subsequently cultured in TSB medium. After 24 h of culturing, the biofilms were collected via ultrasound in 1 mL of PBS for 15 min. The acquired microbiological samples were serially diluted 6-fold (from 10^− 1^ to 10^− 6^) in PBS and spread onto TSA agar plates for incubation at 37 °C in 5 % CO_2_ for 24 h. To determine cell viability, CFU per millimeter of microbiological suspension was counted [16].

Furthermore, the biofilms treated for 24 h were labeled with SYTO9 (LIVE/DEAD Bacterial Viability Kit reagent; BacLight, Invitrogen, Grand Island, NY, USA); live cells appeared green, while dead cells were stained red with propidium iodide. The cells were visualized using epifluorescence microscopy (Nikon Eclipse TE-2000 S, Melville, NY) at 40×magnification. Notably, five random fields in each specimen were selected [16].

### Characterizing biofilm morphology

Scanning electron microscopy (SEM) was applied to assess the structure of biofilms. The samples were diluted twice using PBS and fixed with 2.5 % glutaraldehyde for 4 h. Fixed samples were serially dehydrated with increasing concentrated ethanol solutions from 30 to 100 % and dried using a critical point dryer. Subsequently, samples were coated with gold powder. We obtained micrographs using a scanning electron microscope (Inspect, Hillsboro, OR, USA) [16].

### Analysis of gene expression using quantitative real-time PCR

To further validate the biofilm-associated genes and YycFG pathway modulated by AS*yycG* according to the enrichment results, quantitative real-time PCR was conducted according to the following protocols. Total RNA was extracted and purified with the MasterPure™ RNA Purification Kit (Epicentre Technologies, Epicentre, Madison, WI, USA) from 24-h-treated biofilm samples obtained from the *S. aureus* group, *S. aureus* + H_2_O_2_ group, AS *yycG* group, and AS *yycG* + H_2_O_2_ group following the manufacturer’s instructions. Contaminating genomic DNA was digested and removed with Turbo RNase-free DNase I (Ambion, New York, NY USA) according to the recommended instructions. Next, the purity (A260/A280) and concentration of RNA were determined using a NanoDrop 2000 spectrophotometer (Thermo Scientific, Waltham, MA, USA). The purified RNA was reverse-transcribed to cDNA using random hexamers or gene-specific primers (Table 1) with the RevertAid First Strand cDNA Synthesis Kit (Thermo Scientific). We conducted quantitative real-time polymerase chain reaction (qRT-PCR) assays using the primers listed in Table 1, with the 16Sr RNA gene serving as an internal control [16, 33]. Each sample was analyzed in triplicate, and the threshold cycle values (CT) were quantified.

**Table 1:** Sequences of primers in this study

Primers	sequence 5’-3’ (Forward/Reverse)	Reference
**RT-qPCR**		
*icaA*	5’- GATTATGTAATGTGCTTGGA -3’/	Ref. [30]
	5’- ACTACTGCTGCGTTAATAAT - 3’	
*yycF*	5’ - TGGCGAAAGAAGACATCA -3’/	Ref. [30]
	5’ – AACCCGTTACAAATCCTG- 3’	
*yycG*	5’ - CGGGGCGTTCAAAAGACTTT -3’/	Ref. [30]
	5’ - TCTGAACCTTTGAACACACGT -3’	
*sarA*	5’ - AGATGGCCCTTCTTCAAATG -3’/	This study
	5’ –CCGCAATAATTCTTGTGACG -3’	
*codY*	5’ - GGTGGAGGGGAAAGATTAGG -3’/	This study
	5’ –GCGCGCTTCTTTTTCTACTT -3’	
*16S rRNA*	5’ - GTAGGTGGCAAGCGTTATCC -3’/	Ref. [30]
	5’ –CGCACATCAGCGTCAACA-3’	
**EMSA assay**		
promoter regions of *sarA*	5’ - GCGCAATTTGGTGAAGTTTGATAGATG -3’/	This study
	5’ –GTGATATATAAACCTAGGGCATAAAGTCC -3’	
promoter regions of *icaA*	5’ - CTGAAAATTAATCACACTATGTTACAGG -3’/	This study
	5’ – CTTTACCTACCTTTCGTTAGTTAGGTTG -3’	

The program Primer3 (https://bioinfo.ut.ee/primer3-0.4.0/) was used to design the *sarA* and *codY* primers. For the *sarA* gene, the forward primer targeted starting at nucleotide 194, and the reverse primer targeted starting at nucleotide 429. For the *codY* gene, the forward primer targeted starting at nucleotide 352, and the reverse primer targeted starting at nucleotide 501

### Western blotting

To determine the protein production of YycF and YycG, Western blotting was performed as described below. *S. aureus* ATCC 29,213 and AS*yycG* mutants were cultured to the mid-logarithmic phase (optical density at 600 nm [OD600] = 0.5). Then, we treated the *S. aureus* planktonic cultures with 100 mM H_2_O_2_ for 60 min, washed them and resuspended them in PBS (pH 7.2). The bacterial cells, including the *S. aureus* group, *S. aureus* + H_2_O_2_ group, AS *yycG* group, and AS *yycG* + H_2_O_2_ group, were mechanically disrupted as previously described [31]. For Western blot analysis, equal amounts of protein (30 µg) were mixed with 2X SDS-PAGE Sample Loading Buffer (Sangon Biotech, Shanghai, China) in boiling water for 10 min and loaded on 10 % SDS-PAGE gels (Bio-Rad). Proteins were fractionated and electrotransferred to polyvinylidene fluoride (PVDF) membranes (Biosharp Biotech, Shanghai, China). Membranes were blocked in TSBT buffer (100 mM Tris-HCl, 2.5 mM NaCl) containing 5 % w/v nonfat dry milk at room temperature for 2 h. To measure YycG and YycF production, membranes were incubated with purified YycG- and YycF-specific antibodies (1:500, HuaBio Biotechnology, Hangzhou, China) for 2 h at room temperature, washed in Tris-buffered saline containing 0.1 % Tween 20, and incubated with horseradish peroxidase (HRP)-conjugated goat anti-rabbit secondary antibody (1:5,000) for 2 h at room temperature as previously described [28]. Protein immunoreactive bands were visualized using an Immobilon Western Chemiluminescent kit (Millipore, Billerica, MA, USA). We utilized a Coomassie-stained gel to estimate equal loading of the samples for total bacterial lysis.

### Electrophoretic mobility shift assay (EMSA)

To determine whether the YycF protein could directly bind to the promoter regions of the *sarA* and *icaA* genes, electrophoretic mobility shift assays (EMSAs) were conducted. To generate YycFHis-Tag fusion proteins, the ORF was amplified and restricted. Then, purified products were cloned into digested pET-22b (Novagen) to yield pET-yycG by Huabio Biotech (Hangzhou, China). Plasmids were transformed into *E. coli* BL21. Recombinant proteins were isolated from 500 µL of culture after a 3-h induction with 1 mM IPTG. After cell lysis, recombinant proteins were purified through affinity chromatography on Ni^2+^ NTA agarose (Qiagen) as previously described [43]. The purified protein was visualized via Coomassie staining after SDS-PAGE.

EMSA was used to determine whether the *sarA* or *icaA* gene was directly regulated by YycF as previously described [43] with modifications. A PCR amplicon was generated from *S. aureus* ATCC29213 genomic DNA using primers labeled with the 5’ FAM (Roche) (see Table 1). Before the binding reaction, DNA fragments were purified according to the manufacturer’s instructions (Tiangen Biotech, Beijing, China). Labeled DNA fragments (0.02 pmol) were incubated with increasing amounts of recombinant YycF (0, 20, 40, and 60 pmol) and a 100-fold excess of unlabeled DNA fragments (cold DNA) as a competitor. The reaction mixture (20 µL) contained 50 % glycerinum, labeled DNA fragments, and YycF proteins.

After 20 min incubation at room temperature, samples were loaded on native PAGE gels in 0.5× TBE buffer (44.5 mM Tris-HCl, 44.5 mM boric acid, 1 mM EDTA, pH 8.0). Native PAGE was prepared using 5 × TBE (445 mM Tris-HCl, 445 mM boric acid, 10 mM EDTA, pH 8.0), 30 % Acr-Bis (29:1), 50 % glycerinum, 10 % ammonium persulfate (APS), and N,N,N’,N’-tetramethylethylenediamine (TEMED). Gel electrophoresis was performed at 80 V and 4 °C for 90 min on ice.

### Data analysis

All statistical data were analyzed in SPSS 16.0 (SPSS Inc., Chicago, IL, USA). The Shapiro–Wilk test was used to analyze the distribution of data, and the Bartlett test was used to determine the homogeneity of variances. For parametric testing, we adopted one-way ANOVA to assess the statistical significance of variables followed by the Tukey test. In the data, *P*-values < 0.05 were considered to indicate significant differences.

## Supplementary Information

**Additional file 1:Supplementary Figure 1.** The productions of YycG (A) and YycF (B)were quantified in the groups of *S. aureus, S. aureus + *H2O2, AS *yycG*, and AS *yycG +* H2O2 for Western blotting. **Supplementary Figure 2.** Coomassie-stained gel supporting equal loading of the samples for total bacterial lysis (A). The purified recombinant YycF protein was visualized by Coomassie staining after SDS-PAGE (B).

## Data Availability

The data that support the findings of this study are available from the corresponding author upon reasonable request.

## Abbreviations

*S. aureus*: *Staphylococcus aureus*; AS*yycG*:antisense *yycG* RNA;
PIA: Polysaccharide intercellular adhesion; *S. epidermidis*:*Staphylococcus epidermidis*;
TCSs: two-component signal transduction systems; RR:response regulator;
ASRNA: Antisense RNA; EMSA:electrophoretic mobility shift assay;
EPS: Extracellular polysaccharide substance; PIA:Polysaccharide intercellular adhesion; TSB:Tryptic soy broth; CSP:Competence stimulating peptide;
KEGG: Kyoto Encyclopedia of Genes and Genomes; CV:Crystal violet;
CFU: Colony-forming units; SEM:Scanning electron microscopy;
qRT-PCR: Quantitative real-time polymerase chain reaction; CT:Cycle values;
PVDF: Polyvinylidene fluoride; APS:Ammonium persulfate;
TEMED: tetramethylethylenediamine;
*sarA*: Staphylococcal accessory regulator;
*yycG*: membrane-bound sensor histidine kinase associated with cell wall metabolism.
