# Supplementary material for: Antisense yycG modulates the susceptibility of Staphylococcus aureus to hydrogen peroxide via the sarA
Source: BMC Microbiol. 2021 May 30;21:160. doi: 10.1186/s12866-021-02218-x (PMC8165985; doi:10.1186/s12866-021-02218-x)
Supplement: Supplementary file 1 — Additional file 1:Supplementary Figure 1. The productions of YycG (A) and YycF (B)were quantified in the groups of S. aureus, S. aureus + H2O2, AS yycG, and AS yycG + H2O2 for Western blotting. Supplementary Figure 2. Coomassie-stained gel supporting equal loading of the samples for total bacterial lysis (A). The purified recombinant YycF protein was visualized by Coomassie staining after SDS-PAGE (B). [file 12866_2021_2218_MOESM1_ESM.docx]

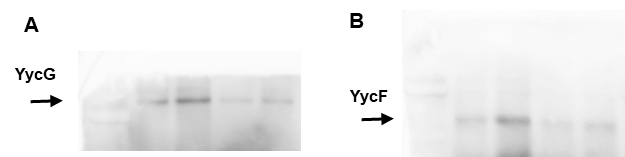


**Supplementary Figure 1.** The productions of YycG (A) and YycF (B)were quantified in the groups of *S. aureus, S. aureus +* H_2_O_2_*,* AS *yycG,* and AS *yycG +* H_2_O_2_ for Western blotting.


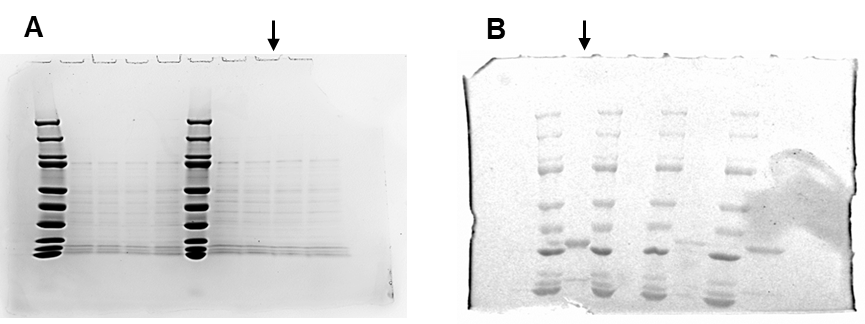


**Supplementary Figure 2.** Coomassie-stained gel supporting equal loading of the samples for total bacterial lysis (A). The purified recombinant YycF protein was visualized by Coomassie staining after SDS-PAGE (B).
